# Supplementary material for: Conditional chemoconnectomics (cCCTomics) as a strategy for efficient and conditional targeting of chemical transmission
Source: eLife. 2024 Apr 30;12:RP91927. doi: 10.7554/eLife.91927 (PMC11060718; doi:10.7554/eLife.91927)
Supplement: Figure 3—source data 3. [file elife-91927-fig3-data3.zip › Figure 3 source data3 part 2.zip!/Figure 3-Source data 3-part2/1053-1-CRISPResso_Report_mpeqg9da.zip!/CRISPResso_on_1053_4/2b.Nucleotide_percentage_quilt_around_sgRNA_ggagcggaatctacaaaacg.pdf]

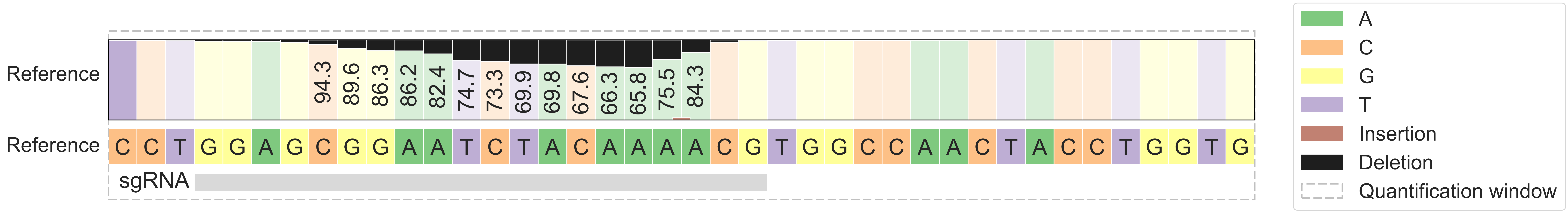

Reference
94.3
89.6
86.3
86.2
82.4
74.7
73.3
69.9
69.8
67.6
66.3
65.8
75.5
84.3
Reference
C C T G G A G C G G A A T C T A C A A A A C G T G G C C A A C T A C C T G G T G
sgRNA
A
C
G
T
Insertion
Deletion
Quantification window
